# Safety and efficacy of intermittent presumptive treatment with sulfadoxine-pyrimethamine using rapid diagnostic test screening and treatment with dihydroartemisinin-piperaquine at the first antenatal care visit (IPTp-SP+): study protocol for a randomized controlled trial

**DOI:** 10.1186/s13063-021-05745-0

**Published:** 2021-11-20

**Authors:** Jean-Bertin Bukasa Kabuya, Matthew M. Ippolito, Jay Sikalima, Clifford Tende, Davies Champo, David Mwakazanga, Anna Marie P. Young, Modest Mulenga, Gershom Chongwe, Christine Manyando

**Affiliations:** 1 Department of Clinical Sciences, Tropical Diseases Research Centre, P.O. Box 71769, Copperbelt Province, Ndola, Zambia; 2 Johns Hopkins University School of Medicine, Baltimore, MD USA; 3 Johns Hopkins Bloomberg School of Public Health, Baltimore, MD USA

**Keywords:** Malaria in pregnancy, Intermittent presumptive therapy, Zambia, Sulfadoxine-pyrimethamine, Dihydroartemisinin-piperaquine

## Abstract

**Background:**

Intermittent preventive treatment in pregnancy (IPTp) with sulfadoxine-pyrimethamine (SP) is recommended by the World Health Organization for the prevention of malaria in pregnancy (MIP)-associated adverse outcomes in high burden areas. However, the efficacy of IPTp-SP has decreased in step with increasing parasite drug resistance. Suitable alternative strategies are needed.

**Methods:**

This is a protocol for a phase IIIb open-label, two-armed randomized controlled superiority trial to assess the safety and efficacy of a hybrid approach to IPTp combining screening and treatment with dihydroartemisinin-piperaquine (DP) to the current IPTp-SP regimen at the first antenatal care clinic visit. Pregnant women without HIV infection and without signs or symptoms of malaria will be randomized to either standard IPTp-SP or hybrid IPTp-SP plus screening and treatment (IPTp-SP+). In the IPTp-SP+ arm, participants who screen positive by rapid diagnostic test for *P. falciparum* will be treated with DP at the first antenatal visit while those who screen negative will receive SP per current guidelines. All participants will be administered SP on days 35 and 63 and will be actively followed biweekly up to day 63 and then monthly until delivery. Infants will be followed until 1 year after delivery. The primary endpoint is incident PCR-confirmed MIP at day 42. Secondary endpoints include incident MIP at other time points, placental malaria, congenital malaria, hemoglobin trends, birth outcomes, and incidence of adverse events in infants up to the first birthday.

**Discussion:**

A hybrid approach to IPTp that combines screening and treatment with an artemisinin-based combination therapy at the first visit with standard IPTp-SP is hypothesized to confer added benefit over IPTp-SP alone in a high malaria transmission area with prevalent SP resistant parasites.

**Trial registration:**

Pan African Clinical Trials Registry 201905721140808. Registered retrospectively on 11 May 2019

**Supplementary Information:**

The online version contains supplementary material available at 10.1186/s13063-021-05745-0.

## Administrative information

Numbers in curly brackets refer to SPIRIT checklist items. The order of the items has been modified to group similar items (see http://www.equator-network.org/reporting-guidelines/spirit-2013-statement-defining-standard-protocol-itemsfor-clinical-trials/). The SPIRIT figure is included as an additional file and the World Health Organization (WHO) Trial Registration Data Set is attached as annex (see supplementary table).

**Table:** 

Title {1}	Safety and Efficacy of Intermittent Presumptive Treatment with Sulfadoxine-Pyrimethamine using Rapid Diagnostic Test Screening and Treatment with Dihydroartemisinin-Piperaquine at the First Antenatal Care Visit (IPTp-SP+): Study Protocol for a Randomized Controlled Trial
Trial Registration {2a and 2b}	PACTR201905721140808 [Pan African Clinical Trials Registry] https://pactr.samrc.ac.za/TrialDisplay.aspx?TrialID=8129 [Registered Retrospectively; May 20, 2019]. https://trialsearch.who.int/Trial2.aspx?TrialID=PACTR201905721140808 All items from the WHO Trial Registration Data Set are listed in the supplementary table
Protocol version {3}	Protocol amendment version 2.2 of October 28, 2019. Revision chronology: V1.0 of December 29, 2017: Original V2.0 of July 13, 2018: revised to incorporate European and Developing Countries Clinical Trial Partnership 2 (EDCTP2)-scientific and ethic committee reviews recommendations: including sample size revision, personal data protection, clarification on study invasive physical procedures to be performed during the trial. V2.1 of February 2019: at the request of local Ethics Review Committee and Regulatory Authorities, statements on analysis population relating to protocol non-adherence and on measurements of the amount of blood to be collected were added to the protocol. V2.2, of October 28, 2019 amendment on inclusion criterion: gestational age changed from 16 to 24 weeks to 16 to 26. Amendment approved by the Ethics Review Committee
Funding {4}	This research is funded by EDCTP2 programme supported by the European Union (EU)
Author details {5a} Roles and responsibilities: sponsor contact information {5b} Roles and responsibilities: sponsor and funder {5c} Roles and responsibilities: committees {5d}	JBK^1^: jeanbertinkabuya@yahoo.com; MMI^2,3^: mippolito@jhu.edu; JS^1^: jsikalima@gmail.com; CT^1^: tendeclifford@gmail.com; DC^1^: dchampo@gmail.com; DM^1^: david.mwakazanga@gmail.com; AMY^2^: ayoung93@jhmi.edu; MM^1^: m.mulenga@hotmail.com; GC^1^: gchongwe@gmail.com; CM^1^: cmanyando@yahoo.com
	Investigator initiated clinical trial: JBK (Principal Investigator), MD, MPSH, Department of Clinical Sciences, Tropical Diseases Research Centre (TDRC), P.O. Box 71769, Ndola, Copperbelt Province, Zambia. Tel.: +260212620737
	This is an investigator initiated clinical trial. Therefore, the funder plays no role in the design of the study including data collection, analysis, and interpretation and manuscript writing.
	Trial oversight is assured by TDRC directorate, trial supervisor/co-investigator (CM) and the Principal Investigator (PI). The PI and co-investigators designed, developed and revised the protocol and a research team composed of all investigators, clinicians, nurses and laboratory technician is responsible for conducting the trial. The maintenance of trial IT system and data entry is done by the data manager. Data verification and monitoring is done by the PI assisted bythe data manager. An external monitor carries out data verification and a Data Safety and Monitoring Board has been established.

^1^Tropical Diseases Research Centre, Ndola, Zambia

^2^Johns Hopkins University School of Medicine, Baltimore, MD, USA

^3^Johns Hopkins Bloomberg School of Public Health, Baltimore, MD, USA

## Background and rationale {6a} {6b}

Malaria poses a major public health risk to pregnant women residing in areas of high *Plasmodium falciparum* transmission [1]. Pregnant women face greater risk of infection and infection-related complications compared to other adults [2]. In high burden areas, malaria in pregnancy (MIP) is usually asymptomatic and is linked to a variety of adverse outcomes including maternal anemia, maternal and perinatal mortality, congenital infection, fetal growth restriction, low birth weight, pre-term birth, and miscarriage [2–5]. The World Health Organization (WHO) therefore recommends intermittent preventive treatment in pregnancy (IPTp) with sulfadoxine-pyrimethamine (SP) to reduce MIP and associated complications in high malaria transmission areas [6].

IPTp relies on contact with patients during scheduled antenatal care clinic (ANC) visits at 4- to 6-week intervals. Treatment doses of antimalarial medication are presumptively administered without prior malaria laboratory testing to clear any potential existing infection and, for a limited post-treatment duration, to protect against subsequent infection [6]. IPTp-SP has been essential in reducing MIP-associated adverse outcomes, with the greatest impact seen when at least three doses of SP are administered throughout a pregnancy [7, 8]. However, the efficacy of SP is waning due to expanding drug resistance [9].

Resistance to SP is caused by point mutations in the genes encoding dihydropteroate synthase (*dhps*) and dihydrofolate reductase (*dhfr*) [10]. Recent studies show that in areas with high prevalence of *dhps* K540E quintuple mutations IPTp-SP effectiveness is reduced but remains associated with increases in birth weight and maternal hemoglobin compared to no IPTp-SP [11, 12]. However, studies from Tanzania and Malawi found that *dhps* A581G sextuple mutations are associated with a greater risk of low birth weight and high peripheral parasitemia, and increased placental infection and inflammation [9, 13]. In Zambia, evidence points to increasing SP resistance and a corresponding increase in breakthrough parasitemia [14, 15]. The prevalences of quintuple and sextuple mutations were estimated to be 17–61% and 2–3%, respectively, in our northern Zambia study site, and were associated with SP failures of 26% and 22% [14, 15].

At present, WHO continues to recommend IPTp-SP in malarious regions where SP resistance remains below certain thresholds while alternative strategies are under investigation [6, 16]. This clinical trial protocol tests the safety and efficacy of a hybrid approach that combines screening and treatment with rapid diagnostic test (RDT) and dihydroartemisinin-piperaquine (DP) at the first antenatal visit in addition to standard IPTp-SP to reduce the incidence of MIP and improve maternal and birth outcomes in a high-transmission area with moderate to high prevalence of *dhps* K540E quintuple mutations.

### Trial objectives {7} {12}

This research protocol is designed to assess the safety and efficacy of a revised IPTp schedule that incorporates a screen-and-treat strategy using RDT and DP at the first antenatal care visit (IPTp-SP+). The research objectives and corresponding endpoints are listed in Table 1.

**Table 1:** Outcomes

Objectives	Outcomes/endpoints
Primary objectives	
To compare the effectiveness of IPTp-SP versus IPTp-SP+ for treating and preventing maternal *P. falciparum* infection To compare the safety and tolerability of IPTp-SP versus IPTp-SP+	• Relative hazard of *P. falciparum* infection diagnosed by PCR at day 42 after randomization (primary outcome) • Relative hazard of *P. falciparum* infection diagnosed by PCR or microscopy at days 14, 28, 35, 42, 63, delivery, and 1-month post-partum in infants • Proportion who experience at least one episode of *P. falciparum* infection by day 14, 28, 35, 42, or 63 • Proportion with treatment or prevention failure at day 42 stratified according to study drug (SP or DP) • Median time to first episode of MIP • Acute, chronic, and prior placental infection at delivery • Medication-related adverse events and serious adverse events until 1-year post-partum (primary outcome)
Secondary objectives	
To compare the effectiveness of IPTp-SP versus IPTp-SP+ for reducing complications of MIP	• Mean birth weight and prevalence of low birth weight (within 72 hours post-partum), neonatal mortality (within 28 days post-partum), congenital malaria diagnosed by PCR, placental malaria diagnosed by histopathology using placental biopsies, maternal anemia (hemoglobin changes at days 14, 28, 42 and 63), congenital anemia, Incidences of pregnancy losses, congenital *P. falciparum* infection
To compare selection for drug resistant parasites between infected individuals in the IPTp-SP and IPTp-SP+ treatment groups	• Relative allele frequencies of genotypic markers of drug resistance in malaria parasites
To measure piperaquine exposure and duration	• Terminal elimination half-life of piperaquine determined by noncompartmental analysis of drug concentrations at 0, 14, 28, 35 and 42 days

*MIP* malaria in pregnancy, *PCR* polymerase chain reaction, *IPTp-SP* intermittent prevent treatment in pregnancy with sulfadoxine-pyrimethamine; *IPTp-SP+*, intermittent prevent treatment in pregnancy with sulfadoxine-pyrimethamine plus screen-and-treat with dihydroartemisinin-piperaquine at first occasion; *SP*, sulfadoxine-pyrimethamine

#### Primary objectives

The two primary objectives are to compare the effectiveness of IPTp-SP with or without screen-and-treat with DP at first antenatal care contact in treating and preventing MIP and to describe the safety profiles of IPTp-SP and IPTp-SP+ including incidence of maternal and perinatal/infant adverse events until 1 year post-partum.

#### Secondary objectives

The secondary objectives include assessment of the relative efficacies of IPTp-SP vs. IPT-SP+ in reducing maternal anemia, improving perinatal outcomes, and preventing placental and congenital malaria. We will also compare the relative impacts of IPTp-SP and IPTp-SP+ on parasite allele frequencies and haplotypes of SP and piperaquine resistance makers among those with incident *P. falciparum* infection.

## Methods

### Study design {8}

This is a phase IIIb open-label randomized controlled superiority trial of standard IPTp-SP vs. IPTp-SP plus screen-and-treat using DP (IPTp-SP+). Participants randomized to the IPTp-SP arm will receive care according to current national guidelines. Participants who are randomized to the IPTp-SP+ arm will receive standard-of-care with the addition of screen-and-treat using RDT and DP at the first ANC visit.

This study protocol has been formatted following SPIRIT reporting guidelines [17].

### Study site {9}

The trial will take place in Nchelenge District, Luapula Province, Zambia. The district is located in the northern wetlands of Zambia alongside Lake Mweru, an area of hyperendemic malaria [18]. Malaria transmission occurs throughout the year. The predominant vector is *Anopheles funestus* which peaks during the dry season (May-September) while both *An. funestus* and *An. gambiae* are found during the rainy season [19]. The population of the district is estimated to be over 296,000 inhabitants consisting mostly of subsistence farmers and fishermen and women. Nchelenge District has one hospital, Saint Paul’s General Hospital (SPGH), twelve rural health centers, and two health posts. The trial will recruit and follow obstetric patients and their offspring at two of the rural health centers and SPGH. SPGH is equipped with an operation theater and labor-and-delivery ward for the provision of essential obstetric services.

### Study population {10} {15}

Pregnant women in the second and third trimesters attending routine ANC visits at the study health facilities will be systematically screened for potential enrollment. Inclusion and exclusion criteria are listed in Table 2. All volunteer pregnant women presenting to the ANC for the first time of their current pregnancy will be assessed for eligibility according to the following criteria: age ≥15 years, estimated gestational age 16–26 weeks, hemoglobin concentration ≥7 g/dL, ability to provide informed consent, residence in the study area with intention to deliver at the health center, no evidence of clinical malaria or other acute illness, and ability to tolerate oral medication. Women will be deemed ineligible for any of the following: infection with human immunodeficiency virus (HIV) or unknown HIV status, prior IPTp-SP or other antimalarial use or antimicrobials with antimalarial activity during the current pregnancy, intolerance to either of the study drugs, existing or prior obstetric complications, prior enrollment in the study or concurrent enrollment in another study, concurrent medications with potential for drug-drug interactions or potentiation of cardiac arrhythmia, presence of chronic illness or other factor deemed likely to influence the pregnancy outcome, or other reason which is judged by the investigator to render the individual unsuitable for study participation.

**Table 2:** Inclusion and exclusion criteria

Inclusion criteria	
• Ability and willingness to provide informed consent^a^	
• Gestational age of 16 to 26 weeks at enrollment	
• Asymptomatic on presentation, with or without a positive RDT test result^b^	
• Age ≥15 years	
• Residence within the study catchment area and no intent to move out of the study catchment area before delivery or to deliver outside of the catchment area	
• Willingness to adhere to all study requirements including to deliver at the health facility	
• Ability to take oral medication	
Exclusion criteria	
• Infection with HIV at enrollment or unknown HIV status^c^	
• History of IPTp-SP or other antimalarial drug use during the current pregnancy	
• History of intolerance or allergic reaction to any of the study drugs	
• History of known pregnancy complications or bad obstetric history including pre-existing illness likely to cause complication of pregnancy such as repeated abortions, stillbirths or eclampsia	
• Hemoglobin concentration <7 g/dL	
• Any significant illness at the time of screening that requires hospitalization, including severe malaria	
• Prior enrollment in the study or concurrent enrollment in another study	
• Treatment with antimicrobials with antimalarial activity within the prior 2 weeks (e.g., clindamycin, azithromycin, tetracycline, clarithromycin, levofloxacin)	
• Concurrent use of medications with potential for drug-drug interaction or potentiation of cardiac arrhythmia^d^	
• History or presence of major illnesses likely to influence pregnancy outcome including hypertension, diabetes mellitus, asthma, epilepsy, renal disease, liver disease, fistula repair, heart disease, or active tuberculosis.	

*HIV*, human immunodeficiency virus; *IPTp-SP*, intermittent preventive treatment with sulfadoxine-pyrimethamine; *RDT*, rapid diagnostic test for *P. falciparum*. ^a^For participants <17 years old, consent will be obtained from the parent or legal guardian and verbal assent will be obtained from the participant. ^b^Asymptomatic defined as absence of fever (temperature <37.5 °C) at baseline and fewer than three of the following symptoms: fever in the past 24 h, headache, weakness/fatigue, myalgia, and arthralgia. ^c^HIV voluntary counseling and testing will be included. We will exclude those on current cotrimoxazole prophylaxis or antiretroviral treatment. ^d^The list of agents includes pentamidine, antiarrhythmic agents (e.g., amiodarone, sotalol), antihistamines (e.g., promethazine), systemic antifungals (ketoconazole, fluconazole, itraconazole), diuretics (e.g., hydrochlorothiazide, furosemide), antipsychotics (haloperidol, thioridazine), antidepressants (imipramine, citalopram, escitalopram), and antiemetics (domperidone, chlorpromazine, ondansetron)

### Randomization and masking {16a} {16b} {16c} {17a} {17b} {23}

The following procedures will be used to ensure an unbiased assessment of treatment safety and efficacy. A computer-generated randomization schedule will be generated by the study statistician prior to the start of the study and kept in a locked cabinet accessible only to the study coordinator who will be responsible for assigning participants to a study treatment arm. The schedule will comprise blocks of varying size to allocate, in a 1:1 ratio, eligible participants to either IPTp-SP or IPTp-SP+. The trial is open-label. However, study investigators, study clinicians, and statisticians are masked to treatment assignment. Investigators will be unmasked after all participants have delivered. A Data and Safety Monitoring Board (DSMB) will be established to review safety data. Statisticians will remain masked until after the DSMB has approved the final analysis plan and the database is locked. All outcome assessors, including laboratory parasitologists and pathologists, will also be masked until the final database is locked.

### Intervention {11a} {11b} {11c}

The intervention will consist of IPTp-SP with or without screen-and-treat using DP at the first ANC visit. Participants randomized to the IPTp-SP+ group who test positive by rapid diagnostic test (RDT) will be given a full treatment course of DP (D-ARTEPP® 40 mg/320 mg three tablets daily for 3 days; Guilin Pharmaceutical) under direct observation (DOT) by a study nurse (day 0) or community health worker (days 1–2). All other participants will be treated with SP at the first visit, including those in the IPTp-SP+ arm with a negative RDT result, and all participants in both groups will be given SP at all subsequent visits (day 35, day 63, then monthly until delivery) under DOT. IPTp-SP will be administered according to national guidelines (G-SCOPE® 1,500 mg sulfadoxine/75 mg pyrimethamine for one dose no fewer than 4 weeks between doses; Guilin Pharmaceutical). Participants will be observed for 1 h post-dose to monitor for vomiting or other adverse reactions. If vomiting occurs within 30 min, the full dose will be readministered. If vomiting occurs after 30 min, half of the total dose will be readministered. In the case of persistent vomiting, the participant will be withdrawn from the study and referred for alternative treatment.

### Clinical procedures {11d} {13} {15} {26a}

**Fig. 1:**
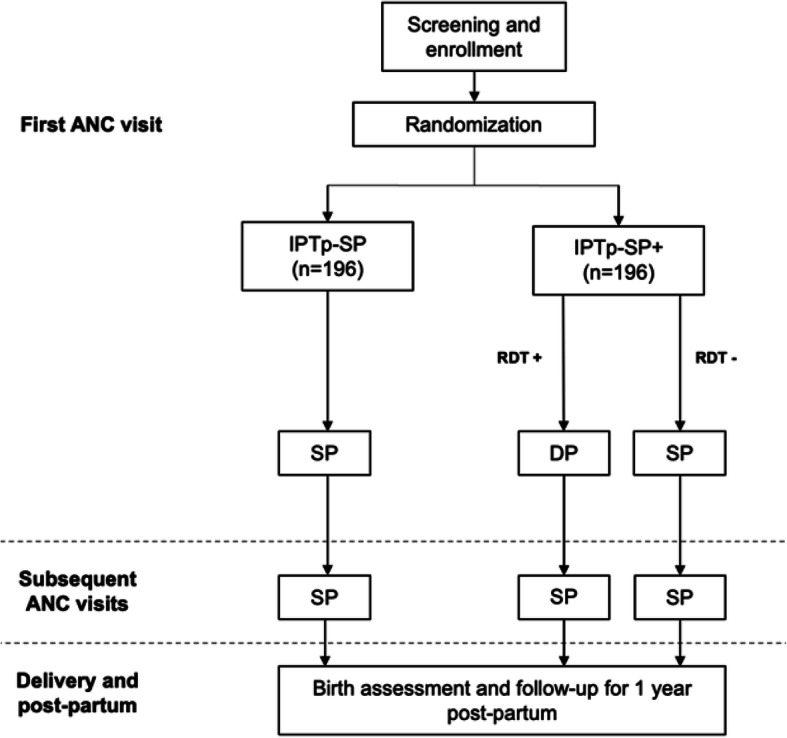
Study flow diagram. *ANC*, antenatal care. *DP*, dihydroartemisinin-piperaquine. *IPTp-SP,* intermittent preventive treatment in pregnancy with sulfadoxine-pyrimethamine with (+) or without screen-and-treat. *RDT*, rapid diagnostic test for *P. falciparum* infection

Study procedures are outlined in Table 3 and the flow of events is shown in Fig. 1. The informed consent will be administered by a study nurse in the local language (Bemba) and/or in English as preferred by the participant. Assent will be obtained from participants <18 years old, and informed consent will be obtained from those ≥18 or the legally authorized representative for minors. If the participant cannot provide a written signature, then a thumbprint will be obtained. After collection of demographic data, provision of informed consent, baseline medical and obstetric history, and physical examination by a study physician including eligibility assessment, participants will be randomized to either the IPTp-SP or IPTp-SP+ treatment groups. All participants will undergo hemoglobin measurement as part of the screening process. On enrollment, fingerstick blood will be drawn for preparation of dried blood spots (DBS) for subsequent molecular testing. In addition, participants randomized to the experimental group (IPTp-SP+) will undergo RDT.

**Table 3:** Schedule of events

	Antenatal period *Study day*									Postpartum period *Study month*			
Event	0	1–2	14	28	35	42	63^a^	Unsch.	Birth	1	6	9	12
Informed consent/oral assent	•												
Medical history	•												
Physical examination, including height, weight, temperature	•		•	•	•	•	•	•	•				
Fetal viability	•		•	•	•	•	•	•	•				
Focused history and examination			•	•	•	•	•	•	•	•	•	•	•
Adverse event evaluation	•	•	•	•	•	•	•	•					
IPTp-SP^b^	•				•		•						
RDT^c^	•												
Treatment with DP^d^	•	•											
DBS for PCR, genotyping	•		•	•	•	•	•	•	•				
Malaria microscopy^e^			•	•	•	•	•	•	•				
Hemoglobin measurement	•		•	•		•	•		•	•			
Blood sample for PK^d^	•		•	•	•	•							
Delivery									•				
Placental biopsy, cord blood									•				
Infant assessment including history and exam, weight, heel stick									•	•	•	•	•

^a^Monthly visits continue until delivery

^b^Includes participants randomized to standard IPTp-SP, participants randomized to IPTp-SP+ who test negative at the first visit, and all participants in both groups at days 35, 63 and then monthly until delivery

^c^Limited to participants randomized to IPTp-SP+

^d^Limited to participants randomized to IPTp-SP+ who test positive by RDT at the first visit

^e^Only participants with signs or symptoms of malaria will undergo microscopy

*DBS* dried blood spot, *DP* dihydroartemisinin-piperaquine, *IPTp-SP* intermittent preventive treatment in pregnancy with sulfadoxine-pyrimethamine, *PCR* polymerase chain reaction, *PK* pharmacokinetics, *RDT* rapid diagnostic test for *P. falciparum* infection

Treatment with either SP or DP will be administered as described above. Participants will return for follow-up on days 14, 28, 35, 42, and 63 and then monthly thereafter until delivery. They will be instructed to report to the clinic for any illness between scheduled visits.

At each scheduled or unscheduled visit, the study clinician will perform an interval history and physical examination, including an updated medication history. During the active follow-up period (up to day 63), DBS will be collected and hemoglobin will be measured. SP will be given on days 35 and 63. Additional doses of SP will be given during monthly visits until delivery with a minimum of 4 weeks between doses and laboratory samples (e.g., blood slide for microscopy, urinary tests, full blood count) will be collected if clinically indicated according to signs and symptoms. Antimalarials or antibiotics with antimalarial activity (e.g., systemic erythromycin, macrolide antibiotics, trimethoprim-sulfamethoxazole or other sulphonamides, tetracyclines, quinolones, clindamycin) will be prohibited during the active follow-up period.

Participants who present during the follow-up period with any signs or symptoms of malaria will undergo testing by thick smear, and DBS will be collected from those with positive microscopy. The signs and symptoms that we will consider indicative of possible clinical malaria include fever, chills, weakness, fatigue, myalgia, arthralgia, headache, anorexia, nausea, vomiting, abdominal pain, or diarrhea. All confirmed uncomplicated malaria cases will be treated with artemether-lumefantrine according to national guidelines (Coartem™ 20 mg/120 mg four tablets taken at 0, 8, 24, 36, 48, and 60 h). Participants who develop severe malaria defined according to WHO criteria will be referred to SPGH for inpatient management [20].

On the day of delivery, the participant and infant will be evaluated as soon as possible after delivery. The birthweight will be measured, and an assessment for congenital abnormalities will be done. Obstetric complications including but not limited to hemorrhage, premature rupture of membranes, cesarean section, and others will be captured as adverse events. Congenital abnormalities, if present, will also be recorded. Thick and thin peripheral blood films, DBS, and hemoglobin measurement will be done on both the participant and infant. In addition, cord blood, placental blood films for thick and thin smear microscopy, placental DBS, and placental biopsy for histopathological analysis will be collected.

Infants will be reassessed at 1, 6, 9, and 12 months postpartum for focused history and physical examination, including interval medication and hospitalization history, growth, and assessment of developmental milestones. At the first monthly visit, hemoglobin measurement and malaria parasitology by thick smear and PCR testing using DBS prepared from heel stick blood will be performed.

### Laboratory procedures {33}

#### Malaria microscopy

Thick blood smears will be stained with 3% Giemsa for 30 min and examined by trained microscopists. Parasite densities will be calculated by counting the number of asexual parasites per 200 leukocytes and the parasite density will be estimated assuming 8000 leukocytes per microliter. A slide will be determined negative after counting 2000 leukocytes. A thin blood smear will be made for species identification and quantification of high parasitemia (>16,000 parasites/μL). Non-falciparum species, gametocytes, and other malaria pigments will be reported but not quantified. Each slide will be read separately by two independent experienced microscopists who will remain masked to treatment assignment.

#### Point-of-care testing

Malaria point-of-care diagnostic testing will be done via *P. falciparum* HRP2 antigen-based RDT (SD BIOLINE, Abbott, IL, USA). Hemoglobin concentrations will be measured using a point-of-care Hb 201 Hemocue® Analyser (Angelholm, Sweden).

#### Placental histopathology

At the time of delivery, a 1-cm^3^ biopsy specimen will be obtained from the maternal-facing side of the placenta. Biopsy specimens will be preserved in 10% neutral buffered formalin and embedded in paraffin wax. Pending histological evaluation, tissue will be kept at 4°C. Paraffin sections 4 mm thick will be stained with hematoxylin and eosin stain. Placental biopsies will be classified according to the following definitions: acute infection (parasites present, malaria pigment absent), chronic infection (both parasites and malaria pigment present), past infection (parasites absent, malaria pigment present), or no infection (both parasites and malaria pigment absent) [21].

#### Molecular assays

DBS collected onto filter paper cards (Whatman® 903 Protein Saver, Sigma-Aldrich) will be allowed to rack-dry overnight and stored individually in opaque sealable plastic bags with desiccant before subsequent use in PCR assays. PCR detection of parasites will be done using cytochrome b. Recurrent episodes will be genotyped to distinguish recrudescence infections from new infections according to standard WHO protocols will be conducted with nested PCR of *merozoite surface protein-1* and -*2* (*msp1*, *msp2*) and glutamate-rich protein (*glurp*) genes for length polymorphisms [22]. Infections will be classified as recrudescent infection if, for each marker, there is at least one identical allele between the initial and recurrent infection. Infections will be classified as reinfections if, for at least one marker, there is a different length polymorphism between the initial and recurrent infection. Infections will be classified as indeterminate in the case of low coverage or missingness due to amplification failure that precludes comparisons at all three genes. Drug resistance markers for artemisinins, SP, piperaquine, and related markers for chloroquine and amodiaquine cross-resistance will be evaluated by molecular inversion probe and amplicon deep sequencing based approaches [23, 24].

#### Plasma piperaquine concentrations

Participants who are randomized to the IPTp-SP+ who screen positive and are administered DP treatment will have an additional 2 mL blood collection for measurement of piperaquine plasma concentrations on days 0, 14, 28, 35, and 42. Piperaquine quantitation will be done using liquid chromatography tandem mass spectrometry as previously described [25]. The terminal elimination half-life will be estimated using noncompartmental analysis, and the effect of age, body surface area, body mass index, and gestational age on drug concentrations will be explored in mixed effects models using WinNonlin software (Certara, Princeton, NJ).

#### Biological specimen storage

Blood slides will be stored in slide boxes. DBS samples will be packaged in zip-lock polythene bags with desiccant and stored and transported in boxes. Placental tissues will be packed in containers with formalin. Samples from study clinics will be taken to the satellite TDRC laboratory in Nchelenge where they will be stored temporarily before shipment to TDRC in Ndola for long-term storage. For participants who consent to future use of specimens, after a 10-year storage period all samples will be destroyed following TDRC standard operating procedures. Biological specimens from participants who do not consent for future use will be destroyed immediately after laboratory analysis. Access to samples by TDRC researchers during the study will be done through the principal investigator, and after the study is completed, it will be done through TDRC management.

### Safety assessments {22}

Drug safety will be monitored at every visit during the course of the study in compliance with International Conference on Harmonization Good Clinical Practice guidelines for adverse events (AEs) and serious adverse events (SAEs) [26]. All expected and unexpected AEs will be collected and reported in trial publications. SAEs will be reported to the sponsor and the ethics committee within 24 working hours of the study staff first becoming aware. Investigators will determine the relationship between safety signals and the study drugs and define outcomes, according to standard classifications [26].

### Sample size calculation {14}

The sample size was determined based on available data and with the objective of achieving a detectable difference of a 50% reduction in the incidence of MIP. In 2019, Nchelenge District recorded 7791 live births (unpublished data). Prior studies in the same population found PCR prevalence of *P. falciparum* parasitemia to be 22–26% in pregnant women [14, 15]. A sample size of 324 pregnant women (*n*=162 per arm) will afford 80% power to detect a 50% difference of effect between study arms with a two-tailed alpha of 0.05. To allow for loss to follow-up of up to 20%, a total of 392 (*n*=196 per arm) will be recruited.

### Data management {18a} {19} {27}

Source data will be recorded using paper case report forms (CRFs) which will then be double-entered into a password-secure electronic database. The CRFs and database will be routinely checked for accuracy by the investigators during the data collection period. The final database will be locked after resolution of all queries. All paper CRFs will be filed and kept in lockable cabinets in offices accessible only to study personnel. At the conclusion of the study, the files will be transferred to the Tropical Diseases Research Centre where they will be stored for a period of 5 years after study completion.

### Participant retention and withdrawal {18b}

To facilitate retention in the study, participants will be issued study visit cards that include the dates of their follow-up visits. We will collect participants’ telephone numbers and physical addresses to allow the study team to contact participants in case of missed visits, advising them to report to the clinic for their scheduled visit within the window period of 3 days. Participants are allowed to leave the study at any time for any reason without any consequences. Participants will be withdrawn from the study only if they withdraw informed consent.

### Statistical analysis {20a} {20b} {20c}

Longitudinal data will be displayed using the nonparametric Kaplan-Meier estimations of the survival function. The primary analysis will be comparison of the 42-day incidence of MIP using multivariate models to estimate the relative hazard between the IPTp-SP and IPTp-SP+ groups according to intention-to-treat (ITT). Testing for proportionality of hazards will be done using formal statistical testing with Schoenfeld residuals as well as visual inspection of the nonparametric survival estimates. For non-proportional hazards, an extended Cox model will be applied. To handle interval censoring due to participants who miss one or more follow-up visits, we will use parametric models for interval-censored survival-time data. The relative hazard will be estimated in parametric models accounting for recurrent events by using a robust variance estimator. For the primary safety analysis, we will compare the proportions of serious and non-serious AEs using logistic regression models and relative hazards of AEs using Cox regression methods as above. Secondary analyses of continuous outcomes (hemoglobin concentration, birthweight) will be examined in linear regression models and in logistic regression models for binary outcomes (prevalence of low birth weight, neonatal mortality, placental malaria, congenital malaria, maternal anemia, congenital anemia, drug resistance allele frequency). Prespecified subgroup analyses will be done according to the following strata: primigravidae, multigravidae, gestational age at enrollment, delivery before or after the day 63 visit, treatment dose on a mg/kg basis, positive *P. falciparum* PCR on enrollment, and presence or absence of quintuple and sextuple mutations. In a secondary analysis, we will compare the proportions of treatment failures and prevention failures according to the antimalarial drug given at the first encounter (SP or DP) in an on-treatment analysis using logistic regression. Treatment failures will be defined as recurrent infections that are determined by genotyping to be recrudescent parasites from the initial infection in participants who tested positive by PCR at the initial visit. Prevention failures will be defined as PCR-confirmed parasitemia in participants who initially tested negative by PCR at the first visit, or who tested positive by PCR at the first visit but who experienced reinfection with a new parasite as determined by genotyping. No interim analysis is planned. Every effort will be made to collect complete data for all participants and minimize missing data. Missing data will be handled using multiple imputation.

### Ethical issues and approval {24} {25} {27}

This protocol has been approved by the European and Developing Countries Clinical Trial Partnership Ethics Review Committee (ERC) and locally by the Tropical Diseases Research Centre ERC, the Zambia Medicine Regulatory Authority, and the National Health Research Ethics Board of the Ministry of Health of the Government of the Republic of Zambia. Any amendment to the study protocol will be submitted to the ERC for approval before its implementation. Data will be de-identified for purposes of publication, data sharing, and secondary analyses. Records containing names or other personal identifiers will be securely stored separately from de-identified data. Expedited or full review, as appropriate, by an ethical review board will be sought for secondary studies. Secondary studies may use stored biological samples from participants who provided consent for future use of blood samples using de-identified data [27].

### Quality assurance and data and safety monitoring board {21a} {23}

Four site visits will be conducted by an independent external monitor who will carry out a minimum of 20% source data verification. A data and safety monitoring board (DSMB) composed of four members will be established. The DSMB will meet quarterly to review progress and provide independent assessments of the quality of the data produced and the safety of study treatments.

## Discussion

### Rationale for this study

Prevention of malaria during pregnancy is important to maternal and child health in endemic areas, but the spread of drug resistant *P. falciparum* has compromised the effectiveness of the current standard approach of IPTp-SP [28–32]. Clinical trials of alternative strategies are therefore needed. This trial protocol describes a hybrid IPTp-SP approach that combines standard IPTp-SP with screening and treatment using DP at the first ANC visit.

There are four justifications to this approach. First, higher density infections that occur early in gestation around the time of the first ANC visit are believed to pose a greater risk to pregnancy than infections that occur later in pregnancy or are lower in density [33–35]. These earlier, higher density infections are often detectable by RDT and are more effectively cleared by curative artemisinin-based combination therapies (ACTs) than by SP, particularly in areas with high quintuple and sextuple mutants. Second, compared to other ACTs, DP provides a period of post-treatment prophylaxis similar to SP owing to piperaquine’s long half-life [36–38]. ACTs with shorter elimination half-lives than DP may be insufficient to bridge the period between ANC visits. Third, screening and treatment during the first visit only—rather than all ANC visits—has the ability of detecting over 50% of all *P. falciparum* infections diagnosed during pregnancy and it is believed to minimize programmatic complexity and resource consumption without sacrificing utility [34]. Mathematical models that compared screening and treatment at the first visit only to screening and treatment at every visit predicted only a marginal difference between the two approaches [33]. Fourth, drug resistance in *P. falciparum* to antifolates is a graduated, rather than all-or-none, phenomenon. Even in areas of prevalent SP resistance genetic markers, IPTp-SP retains a clinically significant level of effectiveness [11]. Some of its effectiveness may be independent of its antimalarial properties. Thus, abandoning SP altogether may not be advisable because it would relinquish a still useful drug while exposing other antimalarials to a greater potential of emerging resistance.

### Alternative strategies to standard IPTp-SP

Various alternatives to standard IPTp-SP are under consideration including the hybrid approach we will test in this protocol [16]. Alternative strategies evaluated in sub-Saharan Africa have so far included IPTp using alternative antimalarials (e.g., DP, artemether-lumefantrine, mefloquine, azithromycin-chloroquine, amodiaquine), intermittent screening and treatment in pregnancy (ISTp) with SP or other agents at one or more ANC visits, or a combination of these approaches [39–47]. Mefloquine (MQ) and DP were shown to be superior to SP for IPTp in reducing incident parasitemia and clinical malaria in areas of widespread SP resistance [42–44]. MQ, however, was less well tolerated and DP was associated with lower birth weight compared to SP [43, 44]. Prior studies also identified the rapid emergence of purported genetic markers of piperaquine resistance within IPTp-DP cohorts [48, 49]. ISTp with artemisinin-based combination therapy at the first ANC visit, in which those who screened negative received no drug at all, was associated with a higher proportion of MIP compared to standard IPTp-SP, attributed to *P. falciparum* parasitemia that went undiagnosed and untreated due to the incomplete sensitivity of RDTs used in screening [44–46].

### Precedent for hybrid models of IPTp-SP

A combined approach of ISTp at the first ANC visit and IPTp-SP at subsequent visits was proposed by WHO as one potential cost-effective MIP prevention strategy to be tested in areas with very high SP resistance [50, 51]. One such approach was implemented as national policy in Tanzania for surveillance purposes in 2014, referred to as “single screen and treat in pregnancy” [52]. Women are screened using an RDT at their first ANC visit, and those who screen positive are treated with either quinine (for women in their first trimester) or an artemisinin-based combination therapy (for women in their second or third trimester) while those who screen negative receive standard IPTp-SP starting from the second trimester. This hybrid strategy was recently evaluated in mathematical models by Walker et al. [33]. The authors modeled five different MIP prevention strategies: IPTp-SP, IPTp-DP, ISTp-DP, and two hybrid strategies of IPTp-SP combined with ISTp-DP either at the first visit only or at every visit. In modeled scenarios that assumed a high frequency of quintuple resistance, the two hybrid strategies performed near equally to each other, while IPTp-DP performed the best and IPTp-SP and IST-DP performed the poorest [33]. The modeled predictions of ISTp and IPTp have been borne out in clinical trials [44, 45], while hybrid strategies have been less closely studied to date.

### Limitations

There are limitations to this study design. A number of previous trials of IPTp included pregnant women who had symptoms of malaria at the time of enrollment. We will exclude women who are symptomatic at the time of their first ANC visit because they must undergo routine clinical evaluation, which includes testing for malaria, and therefore cannot be withheld from malaria screening as would be required if they were randomized to the control group. We will also exclude women who received an antimalarial drug during the current pregnancy to control for contamination bias. These exclusions will reduce the generalizability of the study. Due to the nature of the experimental intervention—ISTp-DP added to standard IPTp-SP—there will be participants who are randomized to the intervention arm but who will receive identical care to that of the comparator arm apart from a single instance of RDT screening. That is, participants randomized to the IPTp-SP+ arm but who screen negative will receive IPTp-SP. This is anticipated to attenuate the observed protective effect of the intervention on the subsequent incidence of MIP due to the mixing of participants who receive DP with those who receive SP at the first ANC visit. However, this has been accounted for in the sample size calculation, and has the advantage of mimicking the real-world application of the strategy to test its effectiveness. We will also account for this by conducting a subgroup analysis comparing IPTp-SP to IPTp-SP+ among participants with a positive *P. falciparum* PCR at the first visit.

### Generating evidence to inform guidelines

Alternative chemopreventive strategies for MIP are urgently needed given the widespread prevalence of SP resistant *P. falciparum* in sub-Saharan Africa. This trial will test a strategy of adding screening and treatment with DP at the first antenatal visit to the standard IPTp-SP to reduce the incidence of MIP and thereby protect against malaria-attributable complications of pregnancy and child development.

## Trial status

This is protocol version 2.2 dated 28 October 2019. Recruitment began on 22 April 2019 and ended on 19 June 2020 with data collection still ongoing. This protocol was not submitted for publication during the active enrollment period due to delays caused by the coronavirus pandemic and medical emergency with prolonged recovery of one of our senior team members.

## Supplementary Information

**Additional file 1.** : Supplementary Table S1. SPIRIT figure

## Abbreviations

ACT: Artemisinin-based combination therapy
AE: Adverse event
ANC: Antenatal care clinic
DBS: Dried blood spot
*dhfr*: Dihydrofolate reductase
*dhps*: Dihydropteroate synthase
DP: Dihydroartemisinin-piperaquine
DSMB: Data and Safety Monitoring Board
ERC: Ethics Review Committee
HIV: Human immunodeficiency virus
IPTp: Intermittent preventive treatment in pregnancy
IPTp-SP: Intermittent preventive treatment in pregnancy with sulfadoxine-pyrimethamine
ISTp: Intermittent screening and treatment in pregnancy
MIP: Malaria in pregnancy
MQ: Mefloquine
RDT: Rapid diagnostic test for *P. falciparum* malaria
SAE: Serious adverse event
SP: Sulfadoxine-pyrimethamine
TDRC: Tropical Diseases Research Centre
WHO: World Health Organization
