# Supplementary material for: Safety and efficacy of intermittent presumptive treatment with sulfadoxine-pyrimethamine using rapid diagnostic test screening and treatment with dihydroartemisinin-piperaquine at the first antenatal care visit (IPTp-SP+): study protocol for a randomized controlled trial
Source: Trials. 2021 Nov 20;22:820. doi: 10.1186/s13063-021-05745-0 (PMC8605457; doi:10.1186/s13063-021-05745-0)
Supplement: Supplementary file 1 — Additional file 1. : Supplementary Table S1. SPIRIT figure [file 13063_2021_5745_MOESM1_ESM.zip › SPIRIT figure.docx]

SPIRIT figure

| SPIRIT figure Schedule of events | | | | | | | | | | | | | | |
| --- | --- | --- | --- | --- | --- | --- | --- | --- | --- | --- | --- | --- | --- | --- |
|  | Study Period | | | | | | | | | | | | | |
|  | Antepartum study period (days) | | | | | | | | | | Postpartum study period *(*month*)* | | | |
| Timepoints | 0 | 1 | 2 | 14 | 28 | 35 | 42 | 63^1^ | UN | Birth | 1 | 6 | 9 | 12 |
| *Procedures* |  |  |  |  |  |  |  |  |  |  |  |  |  |  |
| Informed consent/oral assent | ⚫ |  |  |  |  |  |  |  |  |  |  |  |  |  |
| Medical history | ⚫ |  |  |  |  |  |  |  |  |  |  |  |  |  |
| Physical examination, including height, weight, temperature | ⚫ |  |  | ⚫ | ⚫ | ⚫ | ⚫ | ⚫ | ⚫ | ⚫ |  |  |  |  |
| Fetal viability | ⚫ |  |  | ⚫ | ⚫ | ⚫ | ⚫ | ⚫ | ⚫ | ⚫ |  |  |  |  |
| Focused history and examination |  |  |  | ⚫ | ⚫ | ⚫ | ⚫ | ⚫ | ⚫ | ⚫ | ⚫ | ⚫ | ⚫ | ⚫ |
| Adverse event evaluation | ⚫ | ⚫ | ⚫ | ⚫ | ⚫ | ⚫ | ⚫ | ⚫ | ⚫ | ⚫ | ⚫ | ⚫ | ⚫ | ⚫ |
| *Interventions* |  |  |  |  |  |  |  |  |  |  |  |  |  |  |
| IPTp-SP^2^ | ⚫ |  |  |  |  | ⚫ |  | ⚫ |  |  |  |  |  |  |
| RDT^3^ | ⚫ |  |  |  |  |  |  |  |  |  |  |  |  |  |
| Treatment with DP^4^ | ⚫ | ⚫ | ⚫ |  |  |  |  |  |  |  |  |  |  |  |
| *Assessments* |  |  |  |  |  |  |  |  |  |  |  |  |  |  |
| DBS for PCR, genotyping | ⚫ |  |  | ⚫ | ⚫ | ⚫ | ⚫ | ⚫ | ⚫ | ⚫ |  |  |  |  |
| Malaria microscopy^5^ |  |  |  | ⚫ | ⚫ | ⚫ | ⚫ | ⚫ | ⚫ | ⚫ | ⚫ |  |  |  |
| Hemoglobin measurement | ⚫ |  |  | ⚫ | ⚫ | ⚫ | ⚫ | ⚫ |  | ⚫ | ⚫ |  |  |  |
| Blood sample for PK^4^ | ⚫ |  |  | ⚫ | ⚫ | ⚫ | ⚫ |  |  |  |  |  |  |  |
| Delivery |  |  |  |  |  |  |  |  |  | ⚫ |  |  |  |  |
| Placental biopsy, cord blood |  |  |  |  |  |  |  |  |  |  |  |  |  |  |
| Placental biopsy, cord blood |  |  |  |  |  |  |  |  |  | ⚫ |  |  |  |  |
| Infant assessment including history and exam, weight, heel stick |  |  |  |  |  |  |  |  |  | ⚫ | ⚫ | ⚫ | ⚫ | ⚫ |
| PCR Relative hazard of *P. falciparum* infection | ⚫ |  |  |  |  |  | ⚫ |  |  |  |  |  |  |  |
| PCR or microscopy Relative hazard of *P. falciparum* infection |  |  |  | ⚫ | ⚫ | ⚫ | ⚫ | ⚫ | ⚫ | ⚫ | ⚫ |  |  |  |
| Proportion who experience at least one episode of *P. falciparum* infection |  |  |  | ⚫ | ⚫ | ⚫ | ⚫ | ⚫ | ⚫ | ⚫ |  |  |  |  |
| Proportion with treatment or prevention failure |  |  |  |  |  |  |  | ⚫ | ⚫ |  |  |  |  |  |
| Median time to first episode of MIP |  |  |  | ⚫ | ⚫ | ⚫ | ⚫ | ⚫ |  |  |  |  |  |  |
| Acute, chronic, and prior placental infection at delivery |  |  |  |  |  |  |  |  |  | ⚫ |  |  |  |  |
| Medication-related adverse events |  |  |  |  |  |  | ⚫ | ⚫ |  |  |  |  |  | ⚫ |
| Maternal anemia | ⚫ |  |  | ⚫ | ⚫ | ⚫ | ⚫ | ⚫ |  | ⚫ |  |  |  |  |
| Mean birth weight, Proportion of LBW, neonatal mortality, congenital malaria, placental malaria, congenital anemia, pregnancy losses, |  |  |  |  |  |  |  |  |  | ⚫ | ⚫ |  |  |  |
| Markers of drug resistance | ⚫ |  |  | ⚫ | ⚫ | ⚫ | ⚫ | ⚫ |  |  |  |  |  |  |
| Terminal elimination half-life of piperaquine | ⚫ |  |  | ⚫ | ⚫ | ⚫ | ⚫ | ⚫ |  |  |  |  |  |  |
| ^1^Monthly visits continue until delivery  ^2^Includes participants randomized to standard IPTp-SP, participants randomized to IPTp-SP+ who test negative at the first visit, and all participants in both groups at days 35, 63 and then monthly until delivery  ^3^Limited to participants randomized to IPTp-SP+  ^4^ Limited to participants randomized to IPTp-SP+ who test positive by RDT at the first visit  ^5^Only participants with signs or symptoms of malaria will undergo microscopy  *DBS*, dried blood spot. *DP*, dihydroartemisinin-piperaquine. *IPTp-SP*, intermittent preventive treatment in pregnancy with sulfadoxine-pyrimethamine. *PCR*, polymerase chain reaction. *PK*, pharmacokinetics. *RDT*, rapid diagnostic test for *P. falciparum* infection. *UN,* unscheduled | | | | | | | | | | | | | | |
