# Supplementary material for: Safety and efficacy of intermittent presumptive treatment with sulfadoxine-pyrimethamine using rapid diagnostic test screening and treatment with dihydroartemisinin-piperaquine at the first antenatal care visit (IPTp-SP+): study protocol for a randomized controlled trial
Source: Trials. 2021 Nov 20;22:820. doi: 10.1186/s13063-021-05745-0 (PMC8605457; doi:10.1186/s13063-021-05745-0)
Supplement: Supplementary file 1 — Additional file 1. : Supplementary Table S1. SPIRIT figure [file 13063_2021_5745_MOESM1_ESM.zip › Supplementary Table S1.docx]

Supplementary Table S1

| Supplementary Table WHO Trial Registration Data* | |
| --- | --- |
| Data Category | Information |
| Primary registry and trial identifying number | PACTR201905721140808 [Pan African Clinical Trials Registry] |
| Date of registration in primary registry | May 11, 2019 |
| Source(s) of monetary support | European and Developing Countries Clinical Trial Partnership |
| Primary sponsor | Tropical Diseases Research Centre |
| Contact for public queries | CM, MD, PhD [cmanyando@yahoo.com](mailto:cmanyando@yahoo.com) |
| Contact for scientific queries | CM, MD, PhD Tropical Diseases Research Centre |
| Public title | Intermittent Presumptive Treatment in Pregnancy With Sulfadoxine-Pyrimethamine using Rapid Diagnostic Test Screening And Treatment at First Antenatal Care Visit |
| Scientific title | Safety and Efficacy of Intermittent Presumptive Treatment in Pregnancy With Sulfadoxine-Pyrimethamine using Rapid Diagnostic Test Screening And Treatment at First Antenatal Care Visit |
| Countries of recruitment | Zambia |
| Health condition(s) or problem(s) studied | Malaria, Neonatal Diseases, Pregnancy and Childbirth |
| Intervention(s) | Experimental arm: IPTpSP Plus screening with RDT and treatment using Dihydroartemisinin-Piperaquine (DP) 40/320 mg once daily and/or Sulfadoxine-Pyrimethamine (SP) 500/25 mg once daily |
|  | Control arma: IPTpSP using Sulfadoxine-Pyrimethamine (SP) 500/25 mg |
| Key inclusion and exclusion criteria | Inclusion criteria: Gestational age of 16 to 26 weeks at enrolment; Asymptomatic** on presentation; Hb = 7 g/dL; HIV negative at enrolment; No history of IPTp-SP or antimalarial drug use during the current pregnancy; At least 15 years old; |
|  | Exclusion criteria: Hb<7 g/dl; history of allergic reactions to the study drugs; history of known pregnancy complications or bad obstetric history; history or presence of major illnesses likely to influence pregnancy |
| Study type | Interventional |
| Date of first enrolment | April 22, 2019 |
| Target sample size | 392 |
| Recruitment status | Other |
| Primary outcome(s) | Relative hazard of P. falciparum infection diagnosed by PCR at day 42 after randomization  Medication-related adverse events and serious adverse events until 1-year post-partum |
| Key secondary outcomes | Proportion with treatment or prevention failure at day 42 stratified according to study drug (SP or DP);  Relative hazard of P. falciparum infection diagnosed by PCR or microscopy at days 14, 28, 35, 42, 63, delivery, and 1-month post-partum in infants;  Proportion who experience at least one episode of P. falciparum infection by day 14, 28, 35, 42, or 63;  Median time to first episode of malaria in pregnancy; |
| * <https://trialsearch.who.int/Trial2.aspx?TrialID=PACTR201905721140808>  **Asymptomatic defined as absence of fever (temperature <37.5 °C) at baseline; less than three of the following symptoms: fever in the past 24 h, weakness/fatigue; muscle and/or joint aches, headache | |
